# Deposition and drying dynamics of liquid crystal droplets

**DOI:** 10.1038/ncomms15642

**Published:** 2017-05-30

**Authors:** Zoey S. Davidson, Yongyang Huang, Adam Gross, Angel Martinez, Tim Still, Chao Zhou, Peter J. Collings, Randall D. Kamien, A. G. Yodh

**Affiliations:** 1 Department of Physics & Astronomy, University of Pennsylvania, Philadelphia, Pennsylvania 19104, USA; 2 Department of Electrical & Computer Engineering, Lehigh University, Bethlehem, Pennsylvania 18015, USA; 3 Department of Electrical & Computer Engineering, Bioengineering Program, Lehigh University, Bethlehem, Pennsylvania 18015, USA; 4 Department of Physics & Astronomy, Swarthmore College, Swarthmore, Pennsylvania 19081, USA

## Abstract

Drop drying and deposition phenomena reveal a rich interplay of fundamental science and engineering, give rise to fascinating everyday effects (coffee rings), and influence technologies ranging from printing to genotyping. Here we investigate evaporation dynamics, morphology, and deposition patterns of drying lyotropic chromonic liquid crystal droplets. These drops differ from typical evaporating colloidal drops primarily due to their concentration-dependent isotropic, nematic, and columnar phases. Phase separation occurs during evaporation, and in the process creates surface tension gradients and significant density and viscosity variation within the droplet. As a result, the drying multiphase drops exhibit different convective currents, drop morphologies, and deposition patterns (coffee-rings).

## Abstract (web summary)

When particle-laden drops evaporate, coffee ring patterns form which can affect particle deposition. Here Davidson *et al*. show that unlike previously investigated drops, the flows in drying drops of liquid crystals are driven by an increase in surface tension due to liquid crystal concentration.

Drying drops exhibit a rich phenomenology that depends on the suspended materials, convection and evaporation^1^, surface tension and capillary interactions^2^, contact line pinning and depinning^3^, membrane stretching and bending^4^, Marangoni forces^5^,^6^ and hydrophobicity^7^,^8^. The drying phenomenon thus provides a multi-faceted testing ground for fundamental science and engineering ideas, and insights gained can influence practical applications in printing^9^, genotyping^10^ and other complex assembly and coating schemes^11^,^12^. To date, drying experiments have probed water droplets containing various (mostly small) concentrations of particles^13^,^14^, polymers^15^,^16^, surfactants^17^,^18^, added solvents^19^,^20^ and salts^21^,^22^. These investigations have uncovered fascinating phenomena including coffee rings^13^,^14^,^23^,^24^,^25^, Marangoni flows^5^,^19^,^20^,^26^, electro-wetting effects^27^, complex deposition patterns^21^,^28^ and, in a few cases, formation of concentrated phases very near the drop edge^7^,^29^,^30^,^31^,^32^.

In this contribution we explore the evaporation dynamics, morphology and deposition patterns of drying lyotropic chromonic liquid crystal (LCLC) droplets. These drops differ qualitatively from most others due to their concentration-dependent isotropic, nematic, and columnar liquid crystalline phases in water. As a result, although the LCLC drop starts in its dilute isotropic liquid phase, solute concentration gradients develop and ordered liquid crystal (LC) phases arise in different parts of the drop during evaporation. The concentration profiles, and the formation and separation of liquid crystal phases, in turn, create density, viscosity and surface tension gradients that drive development of convective currents, drop morphologies, and deposition patterns. The phenomenology and understanding thus generated provide insight into how to manipulate and control deposition from an unusual class of drop, for example, drops containing organic mesogens such as dyes, drugs, and biomolecules with potential to form liquid crystal phases in solution^33^,^34^.

Here we investigate these evaporation dynamics and morphology with a model liquid crystal, the dye Sunset Yellow FCF (SSY) in water, whose equilibrium phase behaviour and viscoelastic properties are well understood^35^. A combination of polarized optical microscopy (POM), surface profilometry, and optical coherence microscopy (OCM) permit us to dynamically probe drop morphology, heterogeneous formation of LC phases, and evolving convection currents. In contrast to evaporating DNA or carbon nanotube solutions that sometimes form LC structures very near the drop edges^29^,^30^,^36^, the present experiments reveal formation of distinct nematic and columnar LC domains that span large portions of the drop and trigger unique ‘coffee-ring' phenomenology. The convective flows, the drop morphologies during evaporation, and the final deposition patterns, for example, are heterogeneous and depend strongly on contact angle, SSY concentration and evaporation rate, and the drops are affected by SSY-induced surface tension gradients in counter-intuitive ways.

## Results

### Drying effects observed in isotropic phase

Starting from the earliest stages of the evaporation process, the drying behaviour of isotropic-phase LC droplets differs from that of drying colloidal droplets (for example, coffee drops^23^,^37^). Indeed, although the fluid-glass-air contact line of the LCLC-droplet was pinned and the drop had a spherical cap shape, even at the earliest time scales probed (0.3 s after placing the drop on the slide), the pure radial convective flows towards the drop edge, found in the usual coffee-ring effect, are not observed^23^,^37^. Rather, different and unusual convective flows are found and are described in detail below.

In the earliest drying stage, SSY concentrations throughout the drop remain below that of the isotropic-nematic phase transition, but very soon thereafter SSY mesogens are transported to the drop edge where their concentration builds up. Initially, the SSY suspension has neither translational nor orientational order, and the drop is not birefringent (it appears black when viewed through crossed polarizers). The primary geometrical characteristics that vary during this initial period are the drop height relative to the glass substrate and, to a lesser degree, the contact angle at the drop edge. At the earliest observation times, the drying process deviates from common coffee-ring drop drying behaviour as the SSY concentration increases near the droplet edge. Since the evaporative flux is greatest near the drop edge, convection currents in the drop carry SSY mesogens towards the contact line where the SSY concentration increases and the nematic and columnar phases initially form. The observation that the rate of new phase formation is fastest at the drop edge, where the evaporation rate is also largest, is consistent with observations of Leng *et al*.^38^, which showed enhanced crystallization rates in microfluidic devices with large (controlled) evaporation rates. As evaporation proceeds, a nematic-isotropic phase front, and later a columnar-nematic phase front, propagate radially inward.

**Figure 1:**
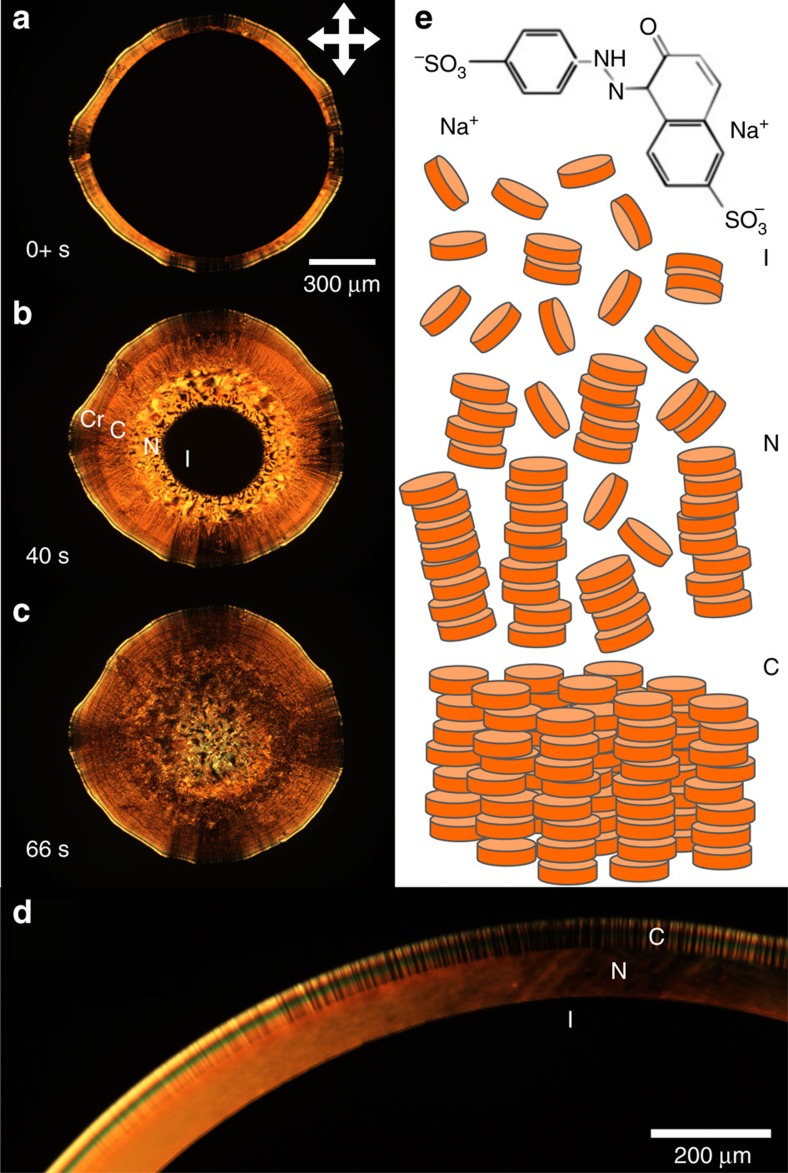
Phases of Sunset Yellow and drop drying. (**a**–**c**) A drying droplet of Sunset Yellow FCF (SSY) on a coverslip under ambient room conditions and with an initial concentration of 15% by weight; the drop is imaged with polarized optical microscopy (POM). (**a**) Recording starts just after the nematic and columnar phases begin to propagate towards the drop center (within ∼30 s of when the drop is placed on the coverslip). (**b**) In the frame taken 40 s later, the four stages (including two LC phases) of the drying process are simultaneously revealed from outer edge to the drop center: crystal (Cr), columnar (C), nematic (N) and isotropic (I). (**c**) The nearly completely dried drop retains much of the order that evolved during the LC phases. (**d**) The highest magnification view shows visual textures common to the I, N and C phases viewed with POM. The darker regions in the drop approximately matching the alignment of the crossed polarizers (crossed double arrows) indicate the director of the LC phases is either parallel or perpendicular to the contact line. We later show the phases are aligned parallel to the contact line. (**e**) Molecular form of SSY salt and schematics of the I, N and C liquid crystal phases.

Ultimately, all water evaporates leaving a polycrystalline ‘coffee-ring' deposit of SSY. During the whole process, texture differences arise between phases within the droplet and are visible in both bright-field and POM. These imaging modalities enable us to distinguish the birefringent nematic and columnar phases from the isotropic phase that remains near the drop center. Under ambient laboratory conditions (20 °C and 40% relative humidity), four stages of the drying process, corresponding to formation of the four complex fluid phases, can be clearly distinguished using POM as shown in Fig. 1 and Supplementary Movie 1.

Before emergence of the anisotropic liquid crystal phases, the drop drying phenomena differs qualitatively from most droplet evaporation studies to date. The first notable difference is the presence of convective flows along the drop-air surface towards the outer contact line where the droplet remains pinned. During evaporation of a pure water droplet, an outward convective flow inside the drop arises because the contact line between the drop edge and the substrate remains pinned at the position of greatest evaporative flux. To compensate for the lost water near the edge, a radially outward flow is established^23^. However, in the liquid crystal droplet, the increased SSY concentration near the drop edge leads to a local increase in the surface tension on the drop surface, and therefore a surface tension gradient arises. The surface tension is larger at the drop edge than near the drop center. The surface tension gradient, in turn, creates a substantial Marangoni flow along the interface towards the air-droplet-glass contact line, accompanied by an inward flow towards the drop center along the droplet-glass interface. The resulting flow pattern produced by the SSY concentration-induced surface tension gradient is thus opposite to Marangoni flows observed in typical water-surfactant drop drying^5^,^39^.

**Figure 2:**
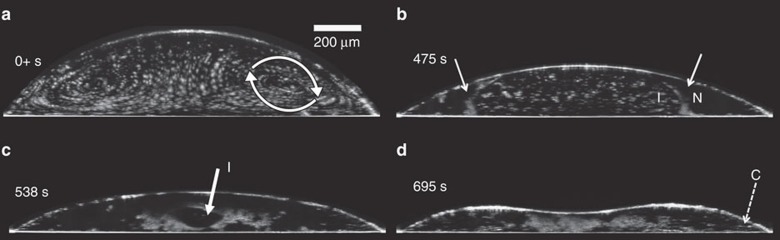
Optical coherence microscopy of fluid flows and LC phases. The drying progression imaged by UHR-OCM. The drop is placed inside a humidity trapping enclosure that slows its drying rate. White spots in the image are micron-diameter polystyrene particles that strongly reflect light and act as tracers of convective fluid flows and LC phase boundaries. Image capture begins within 30 s of placing the drop. (**a**) In the initial drying stage, convective flows move towards the pinned contact line along the drop-air interface and move inward to the drop center along the substrate (see arrows in the frame taken at 0+s). (**b**) At later times, a phase boundary, identified by the arrows, shows that particles in the isotropic phase (I) are prevented from entering the viscous and comparatively dense nematic (N) region that is nucleating from the droplet edge; the particle concentration tends to be large at these phase boundaries. (**c**) Eventually, particles are swept towards the droplet center where they form a shell around a remaining isotropic fluid bubble (arrow, 538 s), and as the region of isotropic phase shrinks to zero volume, the particles irreversibly cluster. The columnar phase first appears in the OCM images as white lines near the droplet edge. These bright lines are not caused by particles; rather, they are cuts through boundaries between domains of varying columnar orientation and thus strongly scatter light. (**d**) The white lines at the edges of the drop in the last frame (695 s, dashed arrow) show the boundaries of columnar phase (C) regions.

Droplet drying was also visualized with a custom ultrahigh resolution spectral domain optical coherence microscopy system (UHR-OCM)^40^. Differences in flow patterns are easily visualized with UHR-OCM by adding 1 μm polystyrene particles to the suspension (Supplementary Note 1 and Supplementary Figs 1 and 2). The patterns are shown in Fig. 2 and in the Supplementary Movies 2–4. Pure circular convective flows are seen from the earliest observed times (at <0.3 s) and persist until the emergence of a nematic phase. The difference in flow circulation direction compared to previous observations with surfactants arises because higher concentrations of SSY at the interface cause the surface tension to increase rather than decrease from its bare value. This behaviour is also observed among many salts and described phenomenologically by the Hofmeister series, which orders anions by their effect on surface tension^41^,^42^. Since SSY has two sulfate groups, which are high in the Hofmeister series, we surmise that the SSY molecules tend to induce a large increase in surface tension. The microscopic causes of these effects in LCLC drops may be related to the unusual amphiphilic structure of SSY, which leads to assemblies of molecules that do not align like conventional surfactant amphiphiles at an interface^43^.

### Liquid crystal phase droplets

**Figure 3:**
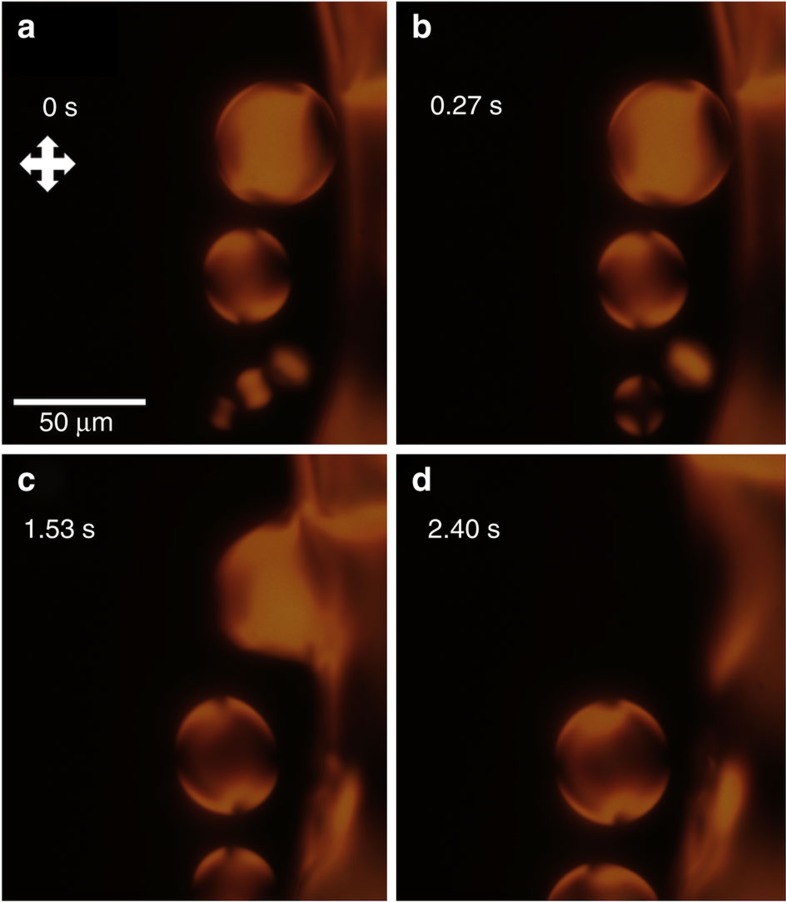
Tactoid formation near the nematic-isotropic phase boundary. At the moving isotropic-nematic phase boundary, nematic tactoids nucleate in the biphasic bulk fluid region near the interface. The tactoids then either coalesce with other tactoids nearby or into the advancing phase boundary. The droplet is viewed by POM and is evaporating under ambient conditions, with an initial concentration of 15% SSY by weight. (**a**,**b**) Between 0 s and 0.27 s, two small nematic tactoids in the lower middle portion of the frame are observed to coalesce into a single larger tactoid. (**c**,**d**) Approximately 1 s later, at 1.53 s, a larger tactoid is seen to coalesce with the phase boundary. The defects that transfer into the bulk annihilate rapidly,that is, before 2.40 s.

After formation of the nematic phase, the isotropic-nematic phase boundary systematically moves towards the drop center from the drop edge. The alternating dark and light regions of the LC phase in Fig. 1, and the light absorption due to linear dichroism (Supplementary Fig. 3), indicate that the average director orientation is parallel to the glass-isotropic-nematic contact line. At higher magnification, we find that the inward moving phase boundary is a biphasic region wherein nematic tactoids (drops with bipolar structure and non-circular cross-section) nucleate in the isotropic region and coalesce into the nematic region as in Fig. 3. This observation of tactoid formation is consistent with findings of small solid/crystal nuclei in the liquid-solid coexistence regions within the microfluidic devices of Leng *et al*.^38^. Because the drying process is out-of-equilibrium and the system is three dimensional, the nucleation and coalescence behaviour in the drop is different from the merging of tactoids observed during cooling of confined two-dmensional (equilibrium) systems^44^. Here topological defects are observed to rapidly annihilate in the continuous nematic region as the phase boundary advances (Supplementary Movie 5).

**Figure 4:**
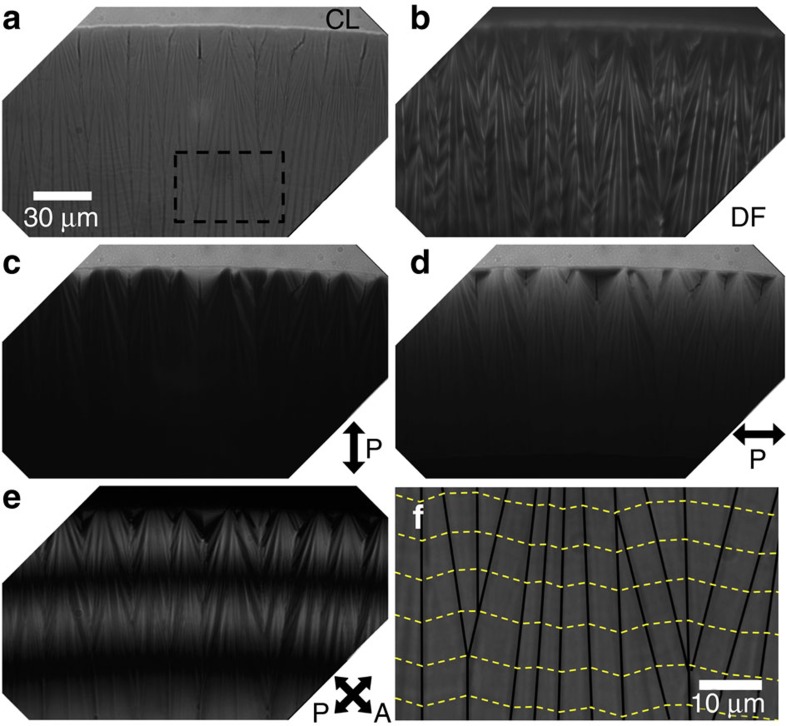
Columnar phase domains at the drop edge. An enclosed drying LCLC droplet with initial concentration of 15% SSY by weight on a glass slide is in the columnar phase near the drop edge: (**a**) Bright field shows dark lines separating columnar domains and the contact line (CL); (**b**) Bright lines in dark field (DF) transmission are regions where light is scattered by sharp changes in the index of refraction caused by disorder; (**c**,**d**) Polarized (P) light transmission with *λ*=470 nm (±15 nm FWHM) is absorbed more when molecular stacking is perpendicular to the light polarization direction. Thus, the data shows columnar alignment is on average tangent to the contact line. (**e**) Crossed-polarized transmission increases contrast between regions with varying columnar alignment. The large black bands are regions where the polarization of the exiting light is extinguished by the analyer (A) due to the birefringence and varying thickness of the sample. (**f**) Schematic of the observed circumferential alignment of the LC from the boxed region in **a**. Dashed yellow lines represent SSY director orientation and thick lines are domain walls of alignment discontinuity. See text for details.

A columnar phase nucleates along the edge of the SSY nematic-substrate contact line before the system completely dries. The texture of the columnar phase depends on the concentration and drying rate of the droplet. When drops are dried in ambient conditions, neighboring regions of columnar phase with slightly different alignment form domain walls (Fig. 4a,b). On-average though, the column orientation is determined to be tangent to the contact line using the linear dichroism effect as in Fig. 4c,d. Tangential alignment is also found in drying drops containing DNA and carbon nanotubes and is due to the lower elastic energy associated with bend distortions compared to splay distortions^29^,^30^, which are present in the SSY systems as well. In addition, albeit with less conclusive evidence, shear stresses near the drop edge can introduce instabilities which unravel/elongate DNA^29^ and which favour reorientation of rod-like structures^30^ parallel to the boundary. Boundary walls separate domains with different orientations that form during the drying process, thereby producing features visually similar to walls observed in other hexagonal columnar lyotropic and discotic systems^29^,^45^. The out-of-equilibrium rapid drying of the system and high viscosity of the columnar phase combine to create kinetically trapped columnar domains that are unable to rearrange to form regions of bend. These walls are apparent in crossed polarized images as in Fig. 4e and close up in Fig. 4f. To further clarify the origin of the columnar domains, we dried the drops extremely slowly in humidity chambers (see Supplementary Figs 4 and 5). In this case, similar domain walls appear within the initially smooth regions of columnar phase. Based on these data and X-ray investigations, which have shown that correlations between molecules and assemblies increase with increasing concentration of the columnar phase^46^,^47^, we suspect that the inter-columnar correlations between molecules create domains of true three-dimensional crystals and thus domain walls are energetically preferred compared to bend deformations.

### Dried LCLC deposits

**Figure 5:**
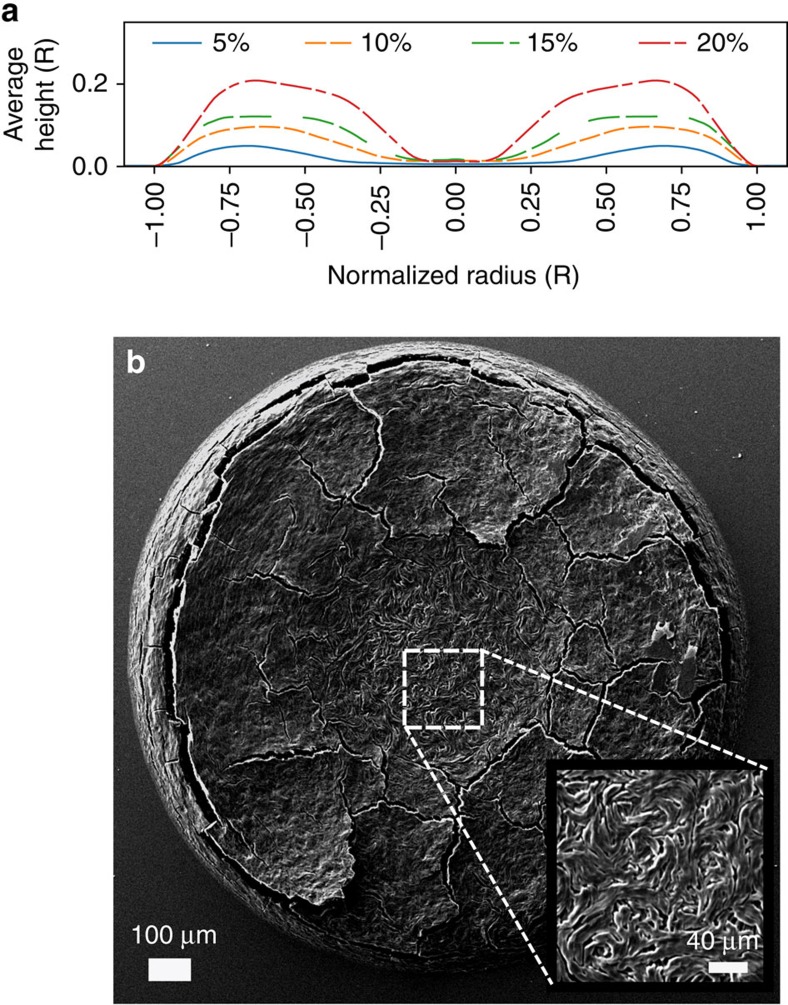
Drop deposits after drying: characterization. (**a**) Profilometry results of dried droplets with varying initial weight concentrations of SSY. Drop size, or more precisely position within the drop, is normalized by the drop radius due to differences in spreading; all drops had a radius of ∼0.5 mm. (**b**) A scanning electron microscope image of a droplet of SSY on a coverslip dried in ambient conditions. The droplet had an initial concentration of 15% SSY by weight. The inner region (inset) of the droplet thins due to the convective flows during drying and locks in the turbulent flows present in the drop just before the transition to the more viscous LC phase.

The final morphology (coffee-ring pattern) of the drop deposit can be complex and depends strongly on the initial concentration of SSY. Similar experiments with droplets confined to cylindrical wells also found a dependence on the initial concentration of solute^48^. Here we measure the surface height profile of the SSY deposit after evaporation with a Zygo surface profilometer, Fig. 5. At low initial concentrations (≤10 wt%), a largely traditional coffee-ring-like effect dominates, depositing the SSY near the drop edge as the contact line recedes. This pattern has only a light covering of SSY molecules near the center of the droplet and a broad rim of SSY molecules near the drop edge (Supplementary Fig. 6). At higher initial SSY concentration (≥10 wt%), however, more SSY is retained in the droplet's central isotropic region, that is, as nematic and columnar phases; along with a surrounding elevated rim, this deposit is akin to a ‘volcano' or a sunken soufflé^48^. This effect occurs when propagation of the nematic phase front is rapid. Throughout the isotropic phase, flows are present, but at higher concentrations, only a small fraction of the SSY has a chance to be deposited near the edges by the flows during drying. This is because the flow into outer regions is blocked by the comparatively large viscosity of the nematic/columnar LC phases and the moving phase boundaries.

Mesogen orientation within the dried deposits was observed with scanning electron microscopy. When drops are allowed to dry quickly in ambient conditions, as in Fig. 5, the central region of the droplet is found to contain small domains, which appear to be turbulent flows frozen in place. Nematic domains arise with various orientations, then merge, and then freeze into the columnar phase. The continued drying and increasing viscosity makes it impossible for the LC to relax to a smooth uniform state during the time before the evaporation finishes. In contrast, the surface of the thicker rim region appears smooth, displaying no signs of trapped local flow. This smoothing is plausible because the SSY near the rim has enough time to anneal to a more homogeneous microstructure. Generally, drops that dried very slowly, that is, drops in humidity controlled chambers, exhibit greater uniformity of molecular orientations over larger regions compared to deposits from drops that dried quickly as shown in Supplementary Fig. 5.

## Discussion

The drying of Sunset Yellow FCF containing droplets exhibits peculiarities unique to lyotropic chromonic liquid crystals. Oriented fluid phases form and move within large regions of the drop throughout the drying process, and SSY concentration gradients lead to Marangoni flows in the isotropic phase. These SSY-induced convective flows circulate opposite to those induced by conventional surfactants. Finally, the initial concentration and drying rate of the SSY solutions affect their final deposition and even the orientation of assemblies in the dried deposit, and are thus revealed to be essential parameters for creating uniform (or non-uniform) material deposits. Since many molecules with LCLC phases are common among dyes and pharmaceuticals, control of their deposition from solution are informed by our findings. Surface roughness resulting from the drying process of LCLCs will affect deposit surface area and light scattering, which are important properties for pharmaceutical and colouring applications, respectively. Furthermore, these observations combined with methods of substrate patterning offer a means to control formation of polarizing and light absorbing films based on LCLCs.

## Methods

### Materials and droplet preparation

SSY-based LCLCs are composed of organic, charged, plank-like molecules that organize in water into column-like mesogenic stacks. The internal structure of these rods depends on a combination of non-covalent electrostatic, excluded volume, hydrophobic, and π–π stacking interactions^46^,^49^. The mesogen assemblies, in turn, organize into nematic or columnar LC phases, depending on temperature and concentration. Under ambient equilibrium conditions, the isotropic—nematic transition occurs at about 28% by weight and the nematic—columnar transition occurs at about 36% by weight^50^. Thus SSY concentration affects two levels of organization: mesogen assembly and LC formation. SSY was purchased from Sigma-Aldrich with 90% purity and was further purified using a precipitation method^51^. SSY solutions of various weight concentrations were prepared with deionized water (*ρ*≥18 MΩ cm).

The SSY droplets were pipetted from vials containing the various initial solution concentrations and were deposited onto clean glass slides or coverslips. Initial contact angles of the drops were observed to vary depending on the SSY concentration and the substrate surface. Generally, as seen in Supplementary Fig. 7, drops with comparatively high initial concentrations of SSY tended to have larger contact angles than lower concentration solutions. Evidently, SSY molecules and associated mesogens adsorbed to the air-water interface cause the surface tension to increase with respect to its bare value, with the largest SSY concentrations causing the largest surface tension increments. We confirmed the surface tension increases by the pendant drop method as reported in Supplementary Fig. 8. In addition, drops at 20% SSY by weight had larger contact angles on coverslips (∼51°) than on glass slides (∼20°).

### Droplet microscopy observations

To connect the observations in the drying droplets to the SSY equilibrium phase diagram, experiments were conducted on an SSY sample at room temperature with a concentration gradient (see Supplementary Note 2). The order of appearance of the phases was the same for both the ‘equilibrium' and drying droplets experiments, and the appearance of the phases themselves was similar for both the ‘equilibrium' and drying droplet experiment.

Typical droplet volumes were 0.2–0.5 μl. Droplet evaporation was observed in both ambient and slow-drying conditions. The latter conditions were achieved by placing droplets in semi-permeable cross-linked polydimethylsiloxane (PDMS) chambers bonded to the substrate and sealed with a cover slip. Evaporation times correspondingly varied, that is, from minutes in ambient conditions to hours in the PDMS chambers. Videos of the evaporation process were captured by transmission optical microscopy with and without crossed polarizers. The use of POM readily permitted assignment of LC phase (for example, isotropic, nematic and columnar) and provided structural information (director configuration). Finally, the predominant orientation of the columns was readily determined by measuring polarized light absorption near the absorption peak of isotropic SSY, *λ*=470 nm (±15 nm FWHM). SSY assemblies exhibit linear dichroism, and since their absorption is greatest for light polarized perpendicular to the liquid crystal director^51^, absorption anisotropy can be utilized to assign director orientation.

### Advanced imaging of droplet drying

OCM employs low coherence interferometry to measure reflected back-scattered signals from different depths within thick samples (droplets)^52^. For flow visualization, the SSY droplets were doped with a very dilute suspension of rinsed micron sized non-functionalized polystyrene particles; note, small numbers of added particles have been shown not to significantly affect the LCLC phases^53^. Time-lapse cross sectional OCM images were acquired over the full course of the drop drying period. Rapid scanning enabled visualization of the internal fluid flow and phase segregation in cross-sectional image planes with an axial resolution of 1.5 μm and transverse resolution of 3.5 μm. Image post-processing (retrieving, cropping and segmentation) was performed with customized software.

The shapes of dried deposits were studied. Droplets were left on slides to dry overnight and were examined using a Zygo New-View 7300 3D Optical Surface profilometer. The surface profilometry combines low coherence white light with a Michelson interferometer in a light microscope operating in reflection-mode to generate a surface height profile with sub-nanometre resolution. Some portions of the dried droplet surface were located at incidence angles too high for illumination light to be reflected back into the objective. The surface height map of these portions were derived by linear interpolation. Drops are azimuthally symmetric over large angles, therefore azimuthal angle averages were computed to derive mean height maps of the dried deposits. Finally, high spatial resolution scanning electron microscopy was used to study the deposits after sputter coating with a thin Au/Pd layer.

### Data availability

The data that support the findings of this study are available from the corresponding author on reasonable request.

## Additional information

**How to cite this article:** Davidson, Z. S. *et al*. Deposition and drying dynamics of liquid crystal droplets. *Nat. Commun.* **8,** 15642 doi: 10.1038/ncomms15642 (2017).

**Publisher's note:** Springer Nature remains neutral with regard to jurisdictional claims in published maps and institutional affiliations.

## Supplementary Material

Supplementary Information Supplementary Figures, Supplementary Notes and Supplementary References

Supplementary Movie 1 This movie at $3\times$ playback speed shows a typical droplet of SSY liquid crystal solution drying throughout the entire evaporation process. Frames from this movie are used in main text, Fig. 1. Drying is carried out under ambient laboratory conditions on a coverslip with an initial SSY concentration of 15\% by weight. Imaging is through crossed polarizers. The droplet diameter is approximately 1.9~mm.

Supplementary Movie 2 This cross-sectional OCM movie shows the initial process of a SSY drop being pipetted onto a coverslip and then beginning to evaporate. Convective flows are initialized within the drop very soon after the drop is pipetted onto the coverslip.

Supplementary Movie 3 OCM cross sectional movie reveals convective flows during drying of a droplet of SSY liquid crystal solution. The movie shows that Marangoni induced convective flows stir micron sized polystyrene particles (white spots) into a flow pattern that suggests the surface tension is higher nearest to the dropletsubstrate contact line. The flow velocity of the particles progressively slows as SSY concentration within the drop increases and corresponding viscosity increases.

Supplementary Movie 4 As the evaporation process continues at higher SSY concentrations, the nematic phase emerges starting from where the droplet contacts the substrate. The movie shows that the nematic-isotropic phase boundary evolves in a way that sweeps most colloidal tracer particles toward the droplet center until, towards the end of the evaporation process, a bubble of isotropic phase remains in the droplet center for a brief period.

Supplementary Movie 5 The nematic phase grows into the isotropic region via a merging with nematic tactoids that form in the nearby isotropic bulk phase. This biphasic region at the moving interface shows tactoids that grow and merge with others nearby (and merge into the bulk nematic phase). This movie includes frames from the main text Fig. 3.
