# Supplementary material for: Deposition and drying dynamics of liquid crystal droplets
Source: Nat Commun. 2017 May 30;8:15642. doi: 10.1038/ncomms15642 (PMC5460001; doi:10.1038/ncomms15642)
Supplement: Supplementary Information — Supplementary Figures, Supplementary Notes and Supplementary References [file ncomms15642-s1.pdf]

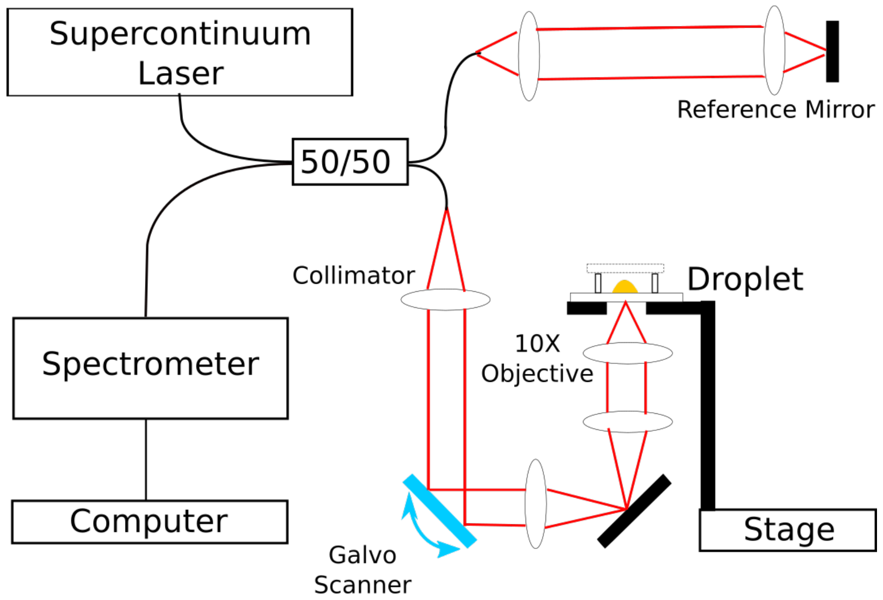

Supplementary Figure 1. **A schematic diagram of the ultrahigh resolution optical coherence microscopy (UHR-OCM) system with an inverted setup.**

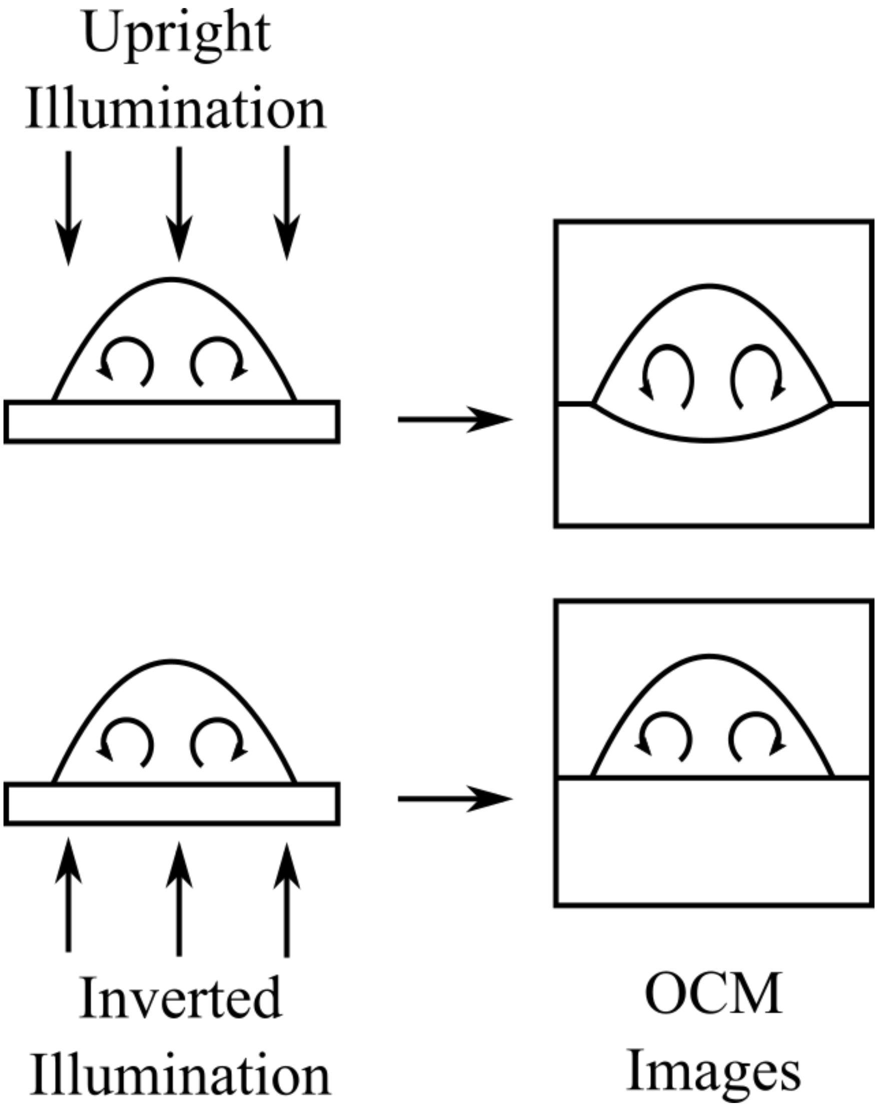

Supplementary Figure 2. **Illumination direction influences the shape of the droplet captured in OCM images.**

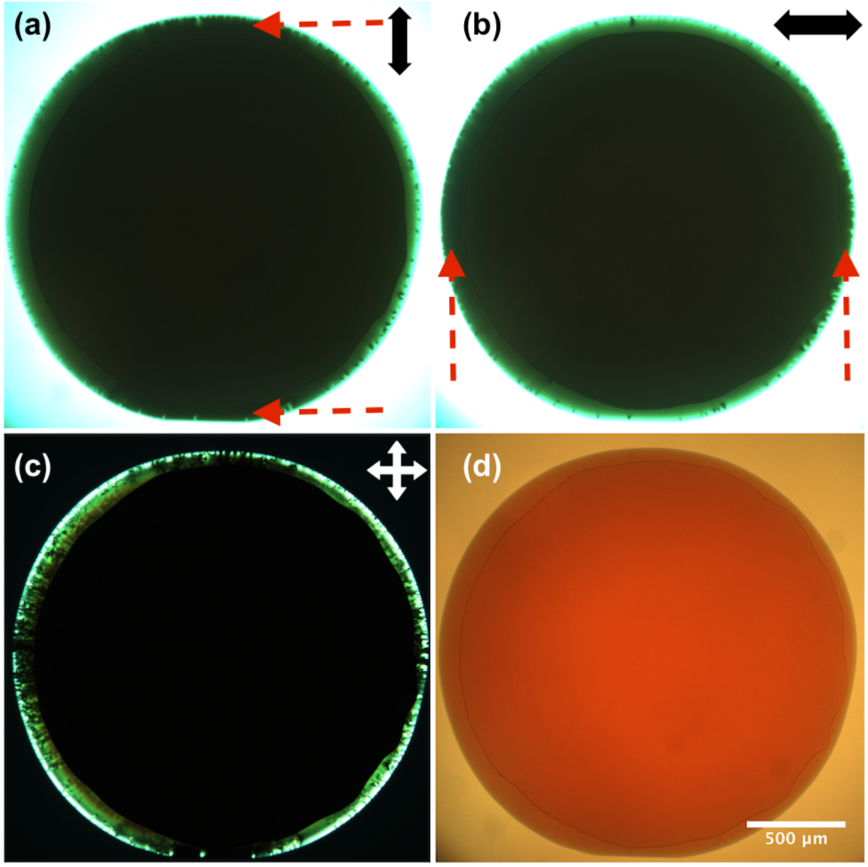

Supplementary Figure 3. **Director alignment in drying drops of Sunset Yellow.** A drying drop of SSY on a glass slide initially with 15 % concentration by weight forms a ring of nematic phase near the contact line with director alignment tangential to the contact line. In the top row, the drop is illuminated with polarized light from a halogen bulb light source and a light filter designed to filter out light with wavelengths greater than 500 nm. Linear dichroism of the nematic phase causes greater absorption of polarized light with orientation indicated by the double black arrows. In (a), the top and bottom regions of the drop indicated by the dashed red arrows are darker than the sides, and in (b), the left and right sides of the drop indicated by dashed red arrows are darker than the top and bottom due to alignment of the nematic director. Crossed polarizers (c) and bright field (d) show similar features to those in the main text (Fig. 1). The columnar and crystalline phases have not yet formed near the drop edges in these images.

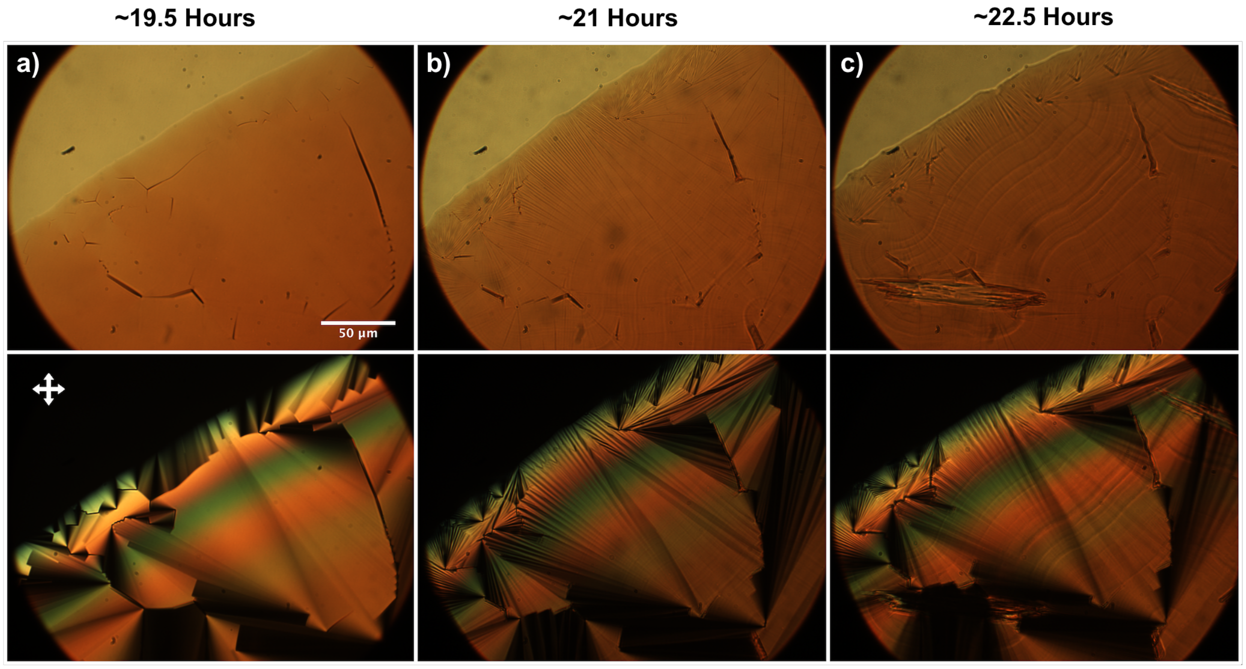

Supplementary Figure 4. **Optical and polarized optical microscopy images of SSY drops in the columnar phase.** Slowly dried drops of SSY on a glass slide show coarsening of the columnar phase as the concentration increases. The top row shows a time series of bright field images and the bottom row shows the corresponding crossed polarized images. Drops were observed once every 1.5 hours. (a) The first observation of the columnar phase was made after $\sim$ 19.5 hours of drying in the humidity chamber. (b) The subsequent observation at $\sim$ 21 hours showed a texture similar to main text Fig. 4, but unlike the main text figure, the texture emerges after the columnar phase is present and not at the nematic-columnar phase boundary. (c) In the last observation at $\sim$ 22.5 hours, crystal phase chunks begin to emerge.

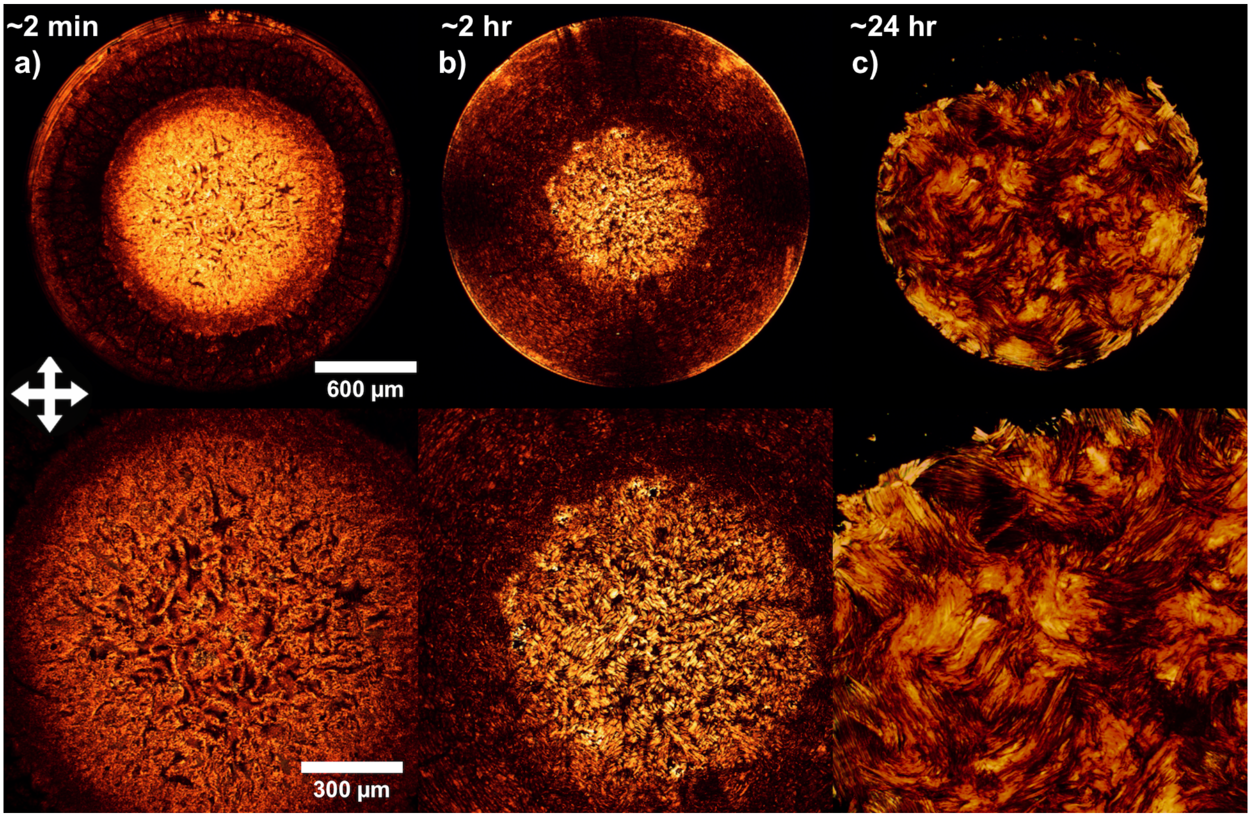

Supplementary Figure 5. **Optical microscopy images of SSY drops dried at different rates.** Fully dried drops of SSY on a coverslip show a morphology dependence on the drying rate of these LCLC drops. The top row shows entire drops each of which had an initial concentration of 15 wt./wt. %. The bottom row are higher magnification images of the central region of the corresponding droplet above. (a) The droplet dried under ambient conditions in approximately two minutes, as in the main text, clearly shows the frozen in place flow pattern. (b) The droplet dried in approximately two hours using a thin walled enclosure shows significant coarsening of domains in the central part of the deposit. (c) At the slowest drying times, over 24 hours, regions of uniform alignment about 100 μm in size are visible. Some of these regions appear to split into thread like domains that may be remnants of domains formed in the columnar phase. Since the sealed drop had much more time in the nematic and columnar phases before it completely dried out, it formed a deposit of more uniform height; hence the lack of the coffee-ring-like deposit pattern.

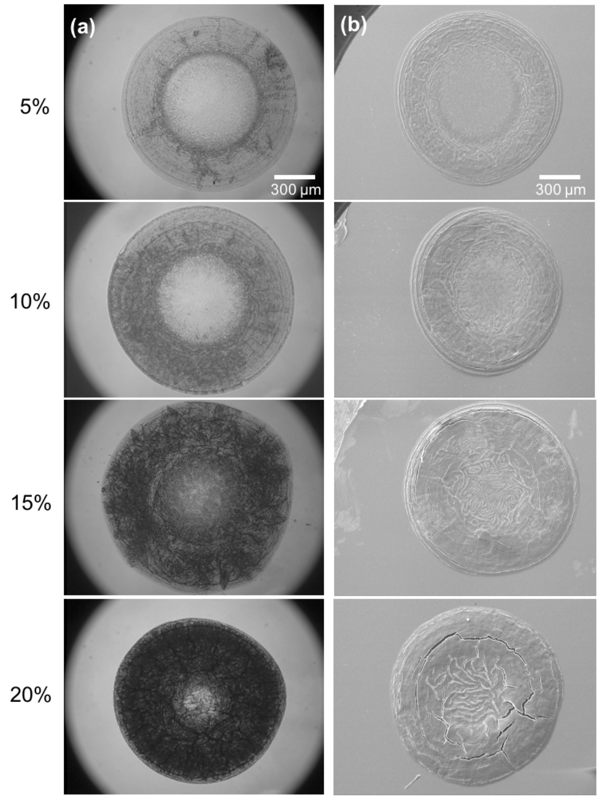

Supplementary Figure 6. **Optical and scanning electron microscopy of dried SSY drops.** Fully dried drops of SSY on a coverslip show a dependence of the dried deposit on initial percent concentration of SSY by weight. Dried deposits are imaged by (a) bright-field microscopy and (b) scanning electron microscopy. Viscosity at higher initial concentrations lessens the influence of coffee ring flows to the point the drop deposit becomes nearly uniform.

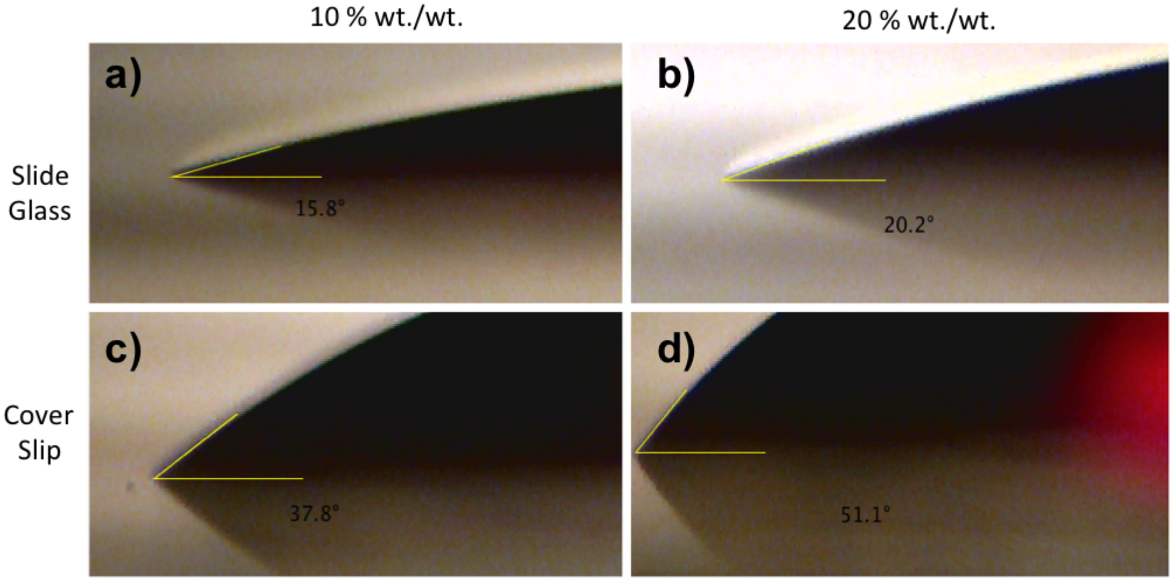

Supplementary Figure 7. **Sunset Yellow contact angles on slides and coverslips.** Measurements of the contact angles of SSY ∼2 µl droplets with varying initial concentrations and on surfaces with varying hydrophobicity. (a,b) Slide glass tends to be more hydrophilic and thus have lower contact angles than (c,d) cover slip surfaces.

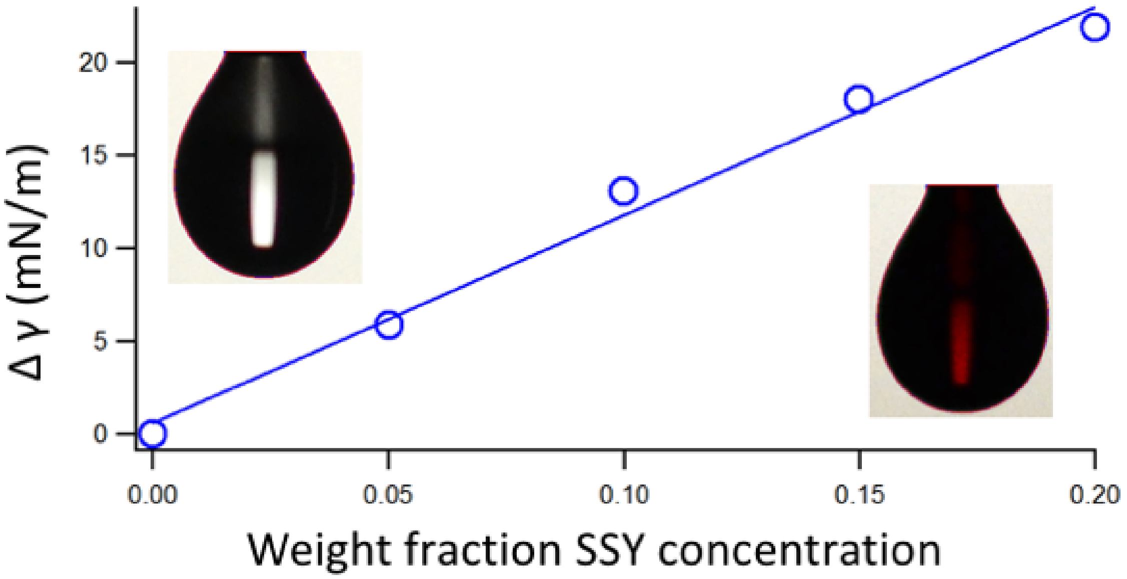

Supplementary Figure 8. Measurements of the surface tension increase of SSY solutions by the pendent drop method. The surface tension increases with increasing SSY concentration. Inset left and right are example images of drops of pure water and 20% by weight respectively. Red lines around the drops are found by the pendent drop software [1].

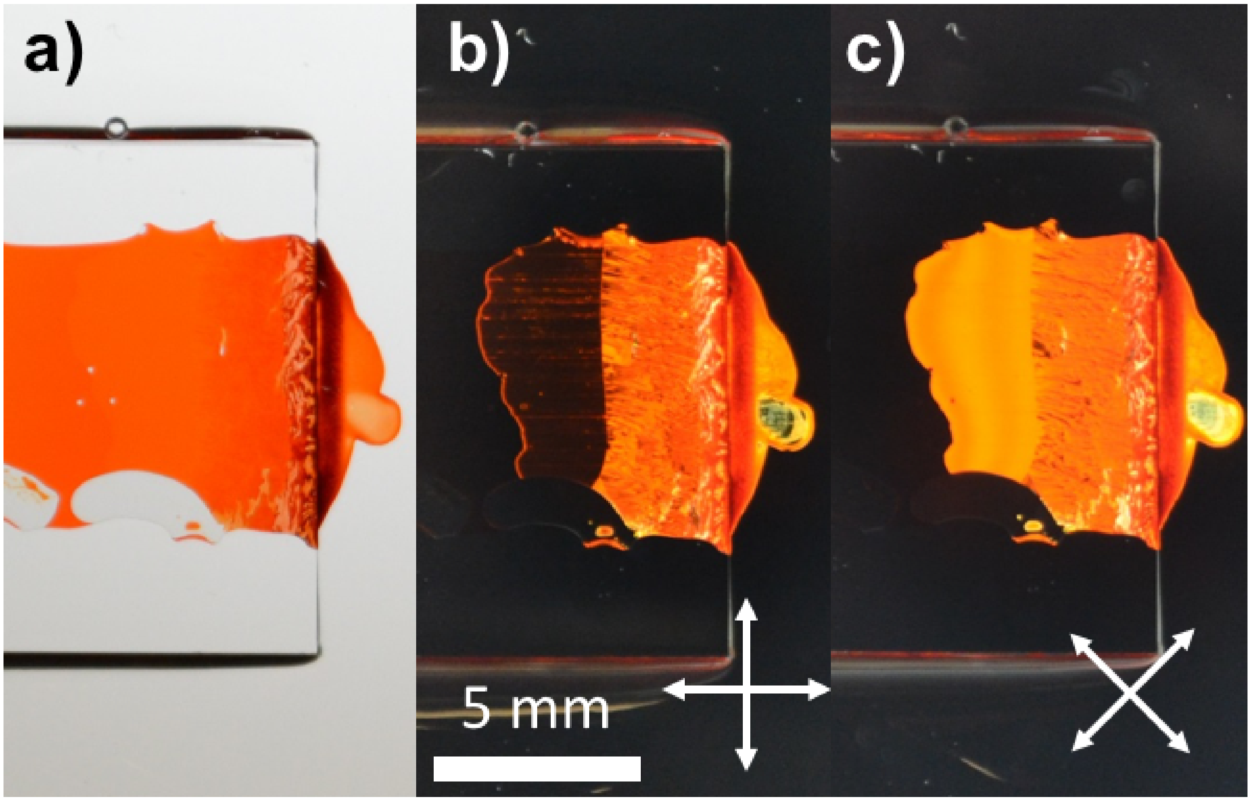

Supplementary Figure 9. **Phase transitions in SSY near equilibrium.** Appearance of SSY at room temperature with a concentration gradient. The SSY concentration increases from left to right. (a) No polarizers, (b) crossed polarizers with one polarizer parallel to the rubbing direction, (c) crossed polarizers with each polarizer at an angle of 45° to the rubbing direction. In all three images, the SSY in the left portion of the cell is in the isotropic phase, the middle portion is in the nematic phase, and the right portion is in the columnar phase.

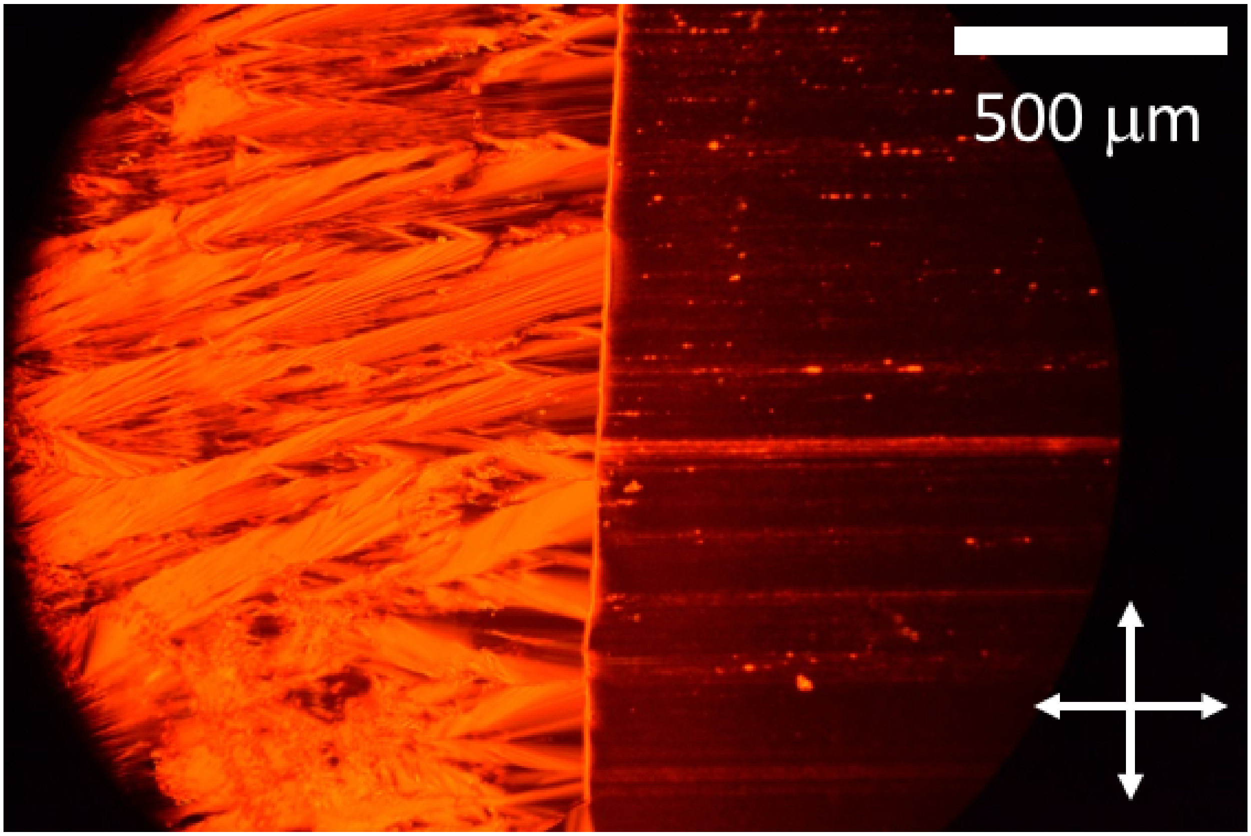

Supplementary Figure 10. **Columnar-Nematic phase border near equilibrium.** The transition between the columnar (left) and nematic (right) phases at room temperature using crossed polarizers and a concentration gradient (higher SSY concentration to the left).

Supplementary Note 1

Optical coherence tomography (OCT) [2–5]is an imaging modality based on low-coherence interferometry. OCT detects back-scattered light within a sample from depths of up to 1-2 millimeters below the sample surface. The reconstructed image has micron-scale resolution in both axial and transverse dimensions. OCT is widely used in biomedical research and in clinical studies, especially in the fields of ophthalmology [6–12] and cardiology [13–17]. Besides biomedical fields [18–20], OCT has been applied in characterization of polymer materials [21, 22] and in studies of art conservation [23–25].

Optical coherence microscopy (OCM) is a high-resolution version of optical coherence tomography [26]. In OCM, a broadband light source, *e.g.* a supercontinuum laser, is utilized to provide an axial resolution as high as $1 - 2$ µm in the sample, and a high numerical aperture (NA) objective is equipped in the sample arm to provide high transverse resolution for imaging. OCM is often employed to observe cellular structure and subcellular features in biomedical samples [27–29]. In our study, OCM is well-suited for droplet imaging because it provides rapid depth-resolved cross-sectional images of the drying drop. Moreover, the high-resolution in both axial and transverse dimensions enables movement tracking of particles at the same scale, *i.e.* $1 - 2$ µm, within the droplet. Previous studies have demonstrated the ability of OCT (OCM) to track particle motion within evaporating drops [30–32].

Supplementary Figure 1 shows a schematic diagram of a custom ultra-high resolution optical coherence microscopy system (UHR-OCM) [27]. A supercontinuum laser (SC-400-4, Fianium Ltd.) is employed to provide a broadband light with a central wavelength of 800 nm and a spectral range of $\sim$ 220 nm, yielding an axial resolution of $\sim$ 1.5 µm in the tissue. Incident light is split 50:50 at the coupler and transmitted to the reference arm and the sample arm separately. A 2D galvanometer (GVS002, Thorlabs) performs transverse scanning of the droplet. A 10x objective is used to image the droplet, yielding a transverse resolution of $\sim$ 3.5 µm. A grating spectrometer with an f-theta lens and a 2048-pixel line scan camera (e2V) is configured to detect back-scattered interferograms at a speed of 20,000 axial scans/s. The sensitivity of this system was measured to be 95dB.

The sample arm of the UHR-OCM system was configured as an inverted microscope, where the illumination beam was shone on the droplet from below (see Supplementary Figure 1). As illustrated in Supplementary Figure 2, with a standard upright illumination configuration, the bottom surface of the droplet (*i.e.* flat cover glass) will be optically distorted, due to a combination of light refraction and increased optical path length within the droplet. With an inverted illumination configuration, OCM imaging beam was shone on the droplet through a flat cover glass, minimizing distortion from light refraction. The acquired OCM images were further scaled in the axial dimension by the index of refraction of the SSY solution, approximated as water ($n = 1.33$), in order to characterize the physical dimension (*i.e.* height) of the droplet.

We probed the earliest stages of the evaporation process with UHR-OCM by initiating image acquisition prior to a LCLC drop being pipetted into a coverslip and PDMS chamber. The pipette tip was positioned directly above the center of the scanning beam, in order to obtain cross-sectional OCM images from the drop center. A coverslip was placed on top of the PDMS chamber immediately after pipetting. 2D scans were performed repeatedly at the same position, with each 2D scan consisting of 600 axial scans. A total of 800 2D scans were obtained, yielding a 24-second imaging time ($\sim$ 33 frames per second). This integration time was sufficient for drop pipetting and observation of initial behavior of the LCLC drop. For visualization, ten consecutive frames were averaged to suppress the speckle noise and improve OCT image quality. From Supplementary Movie 2, we can clearly see that the convective flows are initialized within the LCLC drop immediately after

the drop is pipetted into the chamber. Since the frames are acquired every 0.3 s (0.03 s without averaging), we have shown that the convective flows begin at the earliest stage (or at least a very early stage) of the evaporation process.

A SSY solution drop with 0.2 µl volume was pipetted onto a cover glass (Fisher, 12-548-5E). A humidty chamber enclosure was optionally used to slow down the drying rate. Time-lapse OCM imaging was initiated within $\sim$30 s after pipetting. 2D cross-sectional scanning at the drop center was performed and each 2D scan consisted of 400 axial scans. Transverse image range was adjusted accordingly to cover the whole drop. 2D scans were repeated 30 times at the same cross-section ($\sim$ 0.6 s) and averaged to reduce the speckle noise and improve OCM image quality. Time-lapse OCM data were acquired every 3-4 s, depending on the evaporation rate and the configuration of computer system. Under the typical humidity-controlled condition, the drop dried in approximately 15 minutes. Over 300 OCM slices were obtained to make the time-lapse video of the drop drying progression.

Supplementary Note 2

A concentration gradient of SSY was created in a 20 µm thick cell at room temperature by filling the cell with an SSY solution in the isotropic phase and allowing evaporation of the water to occur very slowly from one side of the sample. The glass surfaces of the cell had been rubbed with a fine abrasive foam to produce alignment of the direction in the nematic phase (see Supplementary Figure 9). Using the SSY equilibrium phase diagram [33], the SSY concentration at the nematic-isotropic transition is 27.8 wt%, and the SSY concentration at the columnar-nematic transition is 35.9 wt%. The appearance of the three phases in this "equilibrium" experiment is similar to the appearance of the three phases in the drying droplet (main text Fig. 1).

An image of the columnar-nematic transition region at higher magnification (see Supplementary Figure 10) reveals that the appearance of the columnar phase in this "equilibrium" experiment is similar to its appearance in the drying droplet (main text Fig. 4 and Supplementary Figure 4).

SUPPLEMENTARY REFERENCES

[1] Daerr, A. & Mogne, A. Pendent drop: An imagej plugin to measure the surface tension from an image of a pendent drop. *Journal of Open Research Software* **4** (2016).

[2] Huang, D. *et al.* Optical coherence tomography. *Science* **254**, 1178–1181 (1991).

[3] Wojtkowski, M. High-speed optical coherence tomography: basics and applications. *Appl. Opt.* **49**, D30 (2010).

[4] Drexler, W. *et al.* Optical coherence tomography today: speed, contrast, and multimodality. *J. Biomed. Opt.* **19**, 071412 (2014).

[5] Fujimoto, J. & Huang, D. Foreword: 25 Years of Optical Coherence Tomography. *Invest. Ophthalmol. Vis. Sci.* **57**, OCTi (2016).

[6] Potsaid, B. *et al.* Ultrahigh speed Spectral / Fourier domain OCT ophthalmic imaging at 70,000 to 312,500 axial scans per second. *Opt. Express* **16**, 15149 (2008).

[7] Nassif, N. A. *et al.* In vivo high-resolution video-rate spectral-domain optical coherence tomography of the human retina and optic nerve. *Opt. Express* **12**, 367 (2004).

[8] Konstantopoulos, A., Hossain, P. & Anderson, D. F. Recent advances in ophthalmic anterior segment imaging: a new era for ophthalmic diagnosis? *Br. J. Ophthalmol.* **91**, 551–557 (2007).

[9] Antcliff, R. J. *et al.* Intravitreal triamcinolone for uveitic cystoid macular edema: An optical coherence tomography study. *Ophthalmology* **108**, 765–772 (2001).

[10] Guedes, V. *et al.* Optical coherence tomography measurement of macular and nerve fiber layer thickness in normal and glaucomatous human eyes. *Ophthalmology* **110**, 177–189 (2003).

[11] Leung, C. K. S. *et al.* Retinal nerve fiber layer imaging with spectral-domain optical coherence tomography a variability and diagnostic performance study. *Ophthalmology* **116**, 1257–1263 (2009).

[12] van Velthoven, M. E. J., Faber, D. J., Verbraak, F. D., van Leeuwen, T. G. & de Smet, M. D. Recent developments in optical coherence tomography for imaging the retina. *Prog. Retin. Eye. Res.* **26**, 57–77 (2007).

[13] Boppart, S. A. *et al.* Noninvasive assessment of the developing xenopus cardiovascular system using optical coherence tomography. *Proc. Nat. Acad. Sci.* **94**, 4256–4261 (1997).

[14] Jang, I. K. *et al.* Visualization of coronary atherosclerotic plaques in patients using optical coherence tomography: comparison with intravascular ultrasound. *J. Am. Coll. Cardiol.* **39**, 604–609 (2002).

[15] Jang, I. K. *et al.* In vivo characterization of coronary atherosclerotic plaque by use of optical coherence tomography. *Circulation* **111**, 1551–1555 (2005).

[16] Tearney, G. J. *et al.* Three-dimensional coronary artery microscopy by intracoronary optical frequency domain imaging. *JACC Cardiovasc. Imaging* **1**, 752–761 (2008).

[17] Suter, M. J. *et al.* Intravascular optical imaging technology for investigating the coronary artery. *JACC Cardiovasc. Imaging* **4**, 1022–1039 (2011).

[18] Zhou, C. *et al.* Integrated Optical Coherence Tomography and Microscopy for Ex Vivo Multiscale Evaluation of Human Breast Tissues. *Cancer Research* **70**, 10071–10079 (2010).

[19] Lee, H.-C. *et al.* Ultrahigh speed spectral-domain optical coherence microscopy. *Biomed. Opt. Express* **4**, 1236 (2013).

[20] Ahsen, O. O. *et al.* Swept source optical coherence microscopy using a 1310 nm VCSEL light source. *Opt. Express* **21**, 18021 (2013).

[21] Stifter, D. Beyond biomedicine: a review of alternative applications and developments for optical coherence tomography. *Appl. Phys. B* **88**, 337–357 (2007).

[22] Stifter, D. *et al.* Dynamic optical studies in materials testing with spectral-domain polarization-sensitive optical coherence tomography. *Opt. Express* **18**, 25712–25725 (2010).

[23] Targowski, P., 243, G., Ra, M. & Wojtkowski, M. Optical Coherence Tomography for Artwork Diagnostics. *Laser Chem.* **2006**, 1–11 (2007).

[24] Liang, H., Lange, R., Peric, B. & Spring, M. Optimum spectral window for imaging of art with optical coherence tomography. *Appl. Phys. B* **111**, 589–602 (2013).

[25] Cheung, C. S., Daniel, J. M. O., Tokurakawa, M., Clarkson, W. A. & Liang, H. High resolution Fourier

domain optical coherence tomography in the 2 μm wavelength range using a broadband supercontinuum source. *Opt. Express* **23**, 1992 (2015).

[26] Aguirre, A. D., Zhou, C., Lee, H.-C., Ahsen, O. O. & Fujimoto, J. G. Optical Coherence Microscopy. In *Optical Coherence Tomography*, Chapter 29, 865–911 (Springer International Publishing, Cham, 2015).

[27] Li, F. *et al.* Label-free evaluation of angiogenic sprouting in microengineered devices using ultrahigh-resolution optical coherence microscopy. *J. Biomed. Opt.* **19**, 16006 (2014).

[28] Srinivasan, V. J., Radhakrishnan, H., Jiang, J. Y., Barry, S. & Cable, A. E. Optical coherence microscopy for deep tissue imaging of the cerebral cortex with intrinsic contrast. *Opt. Express* **20**, 2220–2239 (2012).

[29] Leitgeb, R. A., Villiger, M., Bachmann, A. H., Steinmann, L. & Lasser, T. Extended focus depth for Fourier domain optical coherence microscopy. *Opt. Lett.* **31**, 2450 (2006).

[30] Trantum, J. R. *et al.* Cross-Sectional Tracking of Particle Motion in Evaporating Drops: Flow Fields and Interfacial Accumulation. *Langmuir* **29**, 6221–6231 (2013).

[31] Trantum, J. R., Baglia, M. L., Eagleton, Z. E., Mernaugh, R. L. & Haselton, F. R. Biosensor design based on Marangoni flow in an evaporating drop. *Lab Chip* **14**, 315–324 (2014).

[32] Manukyan, S. *et al.* Imaging internal flows in a drying sessile polymer dispersion drop using Spectral Radar Optical Coherence Tomography (SR-OCT). *J. Colloid Interface Sci.* **395**, 287–293 (2013).

[33] Park, H.-S. *et al.* Self-Assembly of Lyotropic Chromonic Liquid Crystal Sunset Yellow and Effects of Ionic Additives. *J. Phys. Chem. B* **112**, 16307–16319 (2008).
